# E-learning in medical education during COVID-19 pandemic: experiences of a research course at Kenya Medical Training College

**DOI:** 10.1186/s12909-021-03050-7

**Published:** 2021-12-11

**Authors:** Francis Gachanja, Nyawira Mwangi, Wagaki Gicheru

**Affiliations:** 1 Department of Public Health, Kenya Medical Training College, Nairobi, Kenya; 2 Department of Clinical Medicine, Kenya Medical Training College, Nairobi, Kenya; 3 E-learning Department, Kenya Medical Training College, Nairobi, Kenya

**Keywords:** Kenya Medical Training College, E-learning, Research, COVID-19

## Abstract

**Background:**

E-learning has been widely adopted as a teaching and learning approach in medical education internationally. However, its adoption in low- and middle-income countries is still at an infantile stage. The use of e-learning may help to overcome some of the barriers to access to quality education and provide flexible, low-cost, user-centred, and easily updated learning. To address the need for research education during the COVID-19 pandemic, we developed and implemented an e-learning course for students enrolled in higher diploma courses at the Kenya Medical Training College (KMTC). In this paper, we report our experience teaching the online research course in resource-constrained settings to enable other medical educators, students and institutions in similar settings to understand the most appropriate approaches to incorporating e-learning interventions.

**Methods:**

This was a cross-sectional study that reviewed the experiences of learners and lecturers on a research course at Kenya Medical Training College. All higher diploma students admitted to the college in the 2020/21 academic year were invited to take part in the study. We also included all lecturers that were involved in the coordination and facilitation of the course. We analysed qualitative and quantitative data that were collected from the e-learning platform, an online course-evaluation form and reports from course lecturers.

**Results:**

We enrolled 933 students on the online research course. These students had joined 44 higher diploma courses in 11 campuses of the college. The students struggled to complete synchronous e-learning activities on the e-learning platform. Only 53 and 45% of the students were able to complete the pretest and the posttest, respectively. Four themes were identified through a thematic analysis of qualitative data (1) Students gained research competencies (2) Students appreciated the use of diverse e-learning technologies (3) Students felt overwhelmed by the research course (4) Technological challenges reduce the effectiveness of online learning.

**Conclusion:**

Our results suggest that e-learning can be used to teach complex courses, such as research in resource-constrained settings. However, faculty should include more asynchronous e-learning activities to enhance teaching and learning and improve student experiences.

## Background

Electronic learning (e-learning) refers to the use of information, communication and technology (ICT) interventions to deliver, support and enhance learning and teaching [1]. The adoption of e-learning in higher education is supported by the adult learning, cognitive, behavioural and constructivist theories [2, 3]. This approach is popular in medical education because it transcends the boundaries of time and space whilst promoting student-centred, self-directed and collaborative learning [2, 4]. Also, it allows students to create new educational experiences and exercise flexibility in the sequence and pace of learning. However, there is a need for academic leadership to determine when and how to implement e-learning successfully to achieve educational and institutional goals [1, 4].

For medical training institutions in resource-limited settings, the adoption of e-learning may present both opportunities and challenges [5]. E-learning increases the potential for student-teacher engagement and makes it possible to reach a large audience. Also, this approach holds the potential to maximise the use of resources, particularly for educational institutions with few faculty members [4] Current evidence suggests that the adoption of e-learning can lower the cost of education and increase learning opportunities for students in low- and middle-income countries [6, 7]. Indeed, the World Health Organization (WHO) urges LMICs to utilise e-learning as a tool for bridging knowledge and capacity gaps among health workers [8]. Co et al. [9] in a systematic review reported that distance learning pedagogy has the potential to improve learner motivation and performance. Also, inexpensive e-learning solutions can be used to train complex procedures in medical education, such as surgical skills [10]. Therefore, e-learning holds the potential to improve student learning outcomes and experiences, especially in LMICs.

Some of the educational institutions that have adopted e-learning in LMICs have reported a positive response from students and members of faculty [7, 11]. However, challenges may arise when students and faculty transition from face-to-face teaching to e-learning. For example, Abbassi et al. [12] reported that most students in a Pakistani medical training college had a negative attitude towards e-teaching during the COVID-19 lockdown period.

E-learning systems fail because of individual, technological, cultural, environmental, and pedagogical barriers [13, 14]. There is a large digital divide between LMIC as compared to developed countries, which can contribute to poor implementation of e-learning. For example, Makokha et al. [15] attribute the low adoption of e-learning in Kenya to a lack of ICT infrastructure and insufficient skills among faculty members. Co et al. [10] note that even low-cost e-learning solutions may not be viable for students and teachers in economically less-privileged areas. Therefore, educational institutions, faculty members and students may find it difficult to use e-learning systems in resource-constrained settings. For an e-learning system to be effective, students and lecturers must be computer literate and have access to electricity, computing devices and internet connectivity. Even if they meet this criterion, they may face other minor technological challenges. Dhawan [7] points out that faculty and students may face minor challenges such as login difficulties, download errors, and audio-visual problems.

This paper discusses how the college offered the online research course to students while highlighting the lessons learnt. The study aims to track the completion of synchronous and asynchronous e-learning activities in the research course and document students’ and lecturers’ experiences.

## Methods

### Study design

We chose a cross-sectional design that involved the collection of both qualitative and quantitative data.

### Setting

We conducted this study at the Kenya Medical Training College (KMTC). KMTC is a middle-level training institution with campuses in both rural and urban parts of the country. The college provides training to students at certificate, diploma, and higher diploma levels in various health care disciplines. KMTC requires all higher diploma students to undertake a two-week research course in the first semester of their studies.

KMTC has traditionally used face-to-face and blended pedagogical approaches in training medical professionals. However, on March 11, 2020, the World Health Organization declared COVID-19 a pandemic [9]. In response, the college unexpectedly transitioned to online learning through the Moodle e-learning platform to mitigate the spread of the disease and comply with government-imposed restrictions.

To implement the transition from face-to-face teaching to e-learning of a research course, the college organised the faculty on the course into two multidisciplinary teams. The first team organised teaching and learning activities, while the second monitored learner participation and provided student support. We set up a WhatsApp group, which facilitated real-time deliberations between faculty on both teams. Also, faculty and students communicated through text, phone calls, emails, and communication tools that were provided by the Moodle learning management system.

The first team uploaded learning content on the KMTC learning management system. This content comprised text-based material, pre-recorded videos, downloadable resources, and assessment quizzes. Further, members of faculty held online classes using the zoom video conferencing software. All online classes were recorded and later uploaded to the e-learning platform. Therefore, students who did not attend the online classes were able to access the recorded sessions thereafter.

Further, we required students to complete a pretest, a posttest, and a peer-graded written assignment. We trained students on how to assess their peers and provided a grading rubric to standardise the assessment. Also, we required students to submit the written assignment and assess their peers through the Moodle e-learning platform. However, the peer assessment activity was asynchronous, and students were allowed 3 days to complete the activity. The pretest and posttest were conducted online synchronously.

### Participants

We invited all first-year higher diploma students that were enrolled in the online research course to take part in the study (*n* = 933). Learners who joined the course on the Moodle LMS after the two-week teaching period had elapsed were excluded. However, they were allowed access to material on the platform.

We also invited all lecturers who were involved in the teaching and coordination of the course.

### Data collection

We obtained data on student completion of online learning activities from the Moodle e-learning platform. In addition, we used an end-of-course evaluation questionnaire with closed and open-ended questions to collect the data. The quantitative section of the questionnaire examined the quality and the value of the course to the student. The qualitative section focused on students’ experiences in the e-learning course. We also extracted data from the summative reports provided by the course organisers and the meeting notes from the end-of-course meeting between students and lecturers.

### Data analysis

Statistical analysis of quantitative data was performed using R statistical software version 4.04. We summarised the data in counts, frequencies, and percentages.

We used thematic analysis to explore and generate key themes in responses to the open-ended questions. We used a systematic approach that involved familiarizing ourselves with the data, searching for themes, reviewing emerging themes, defining themes and reporting.

## Results

**Fig. 1:**
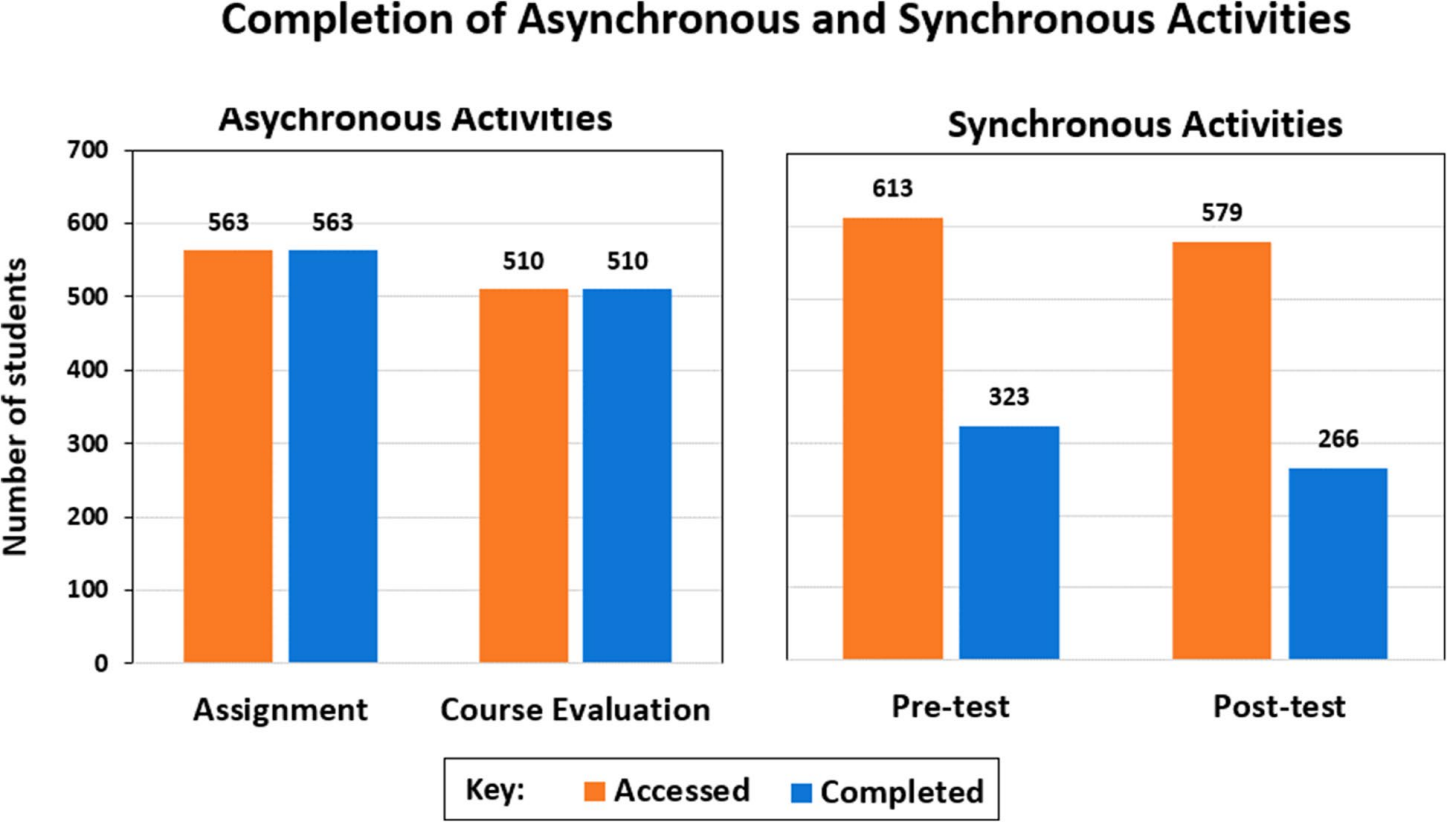
Completion of asynchronous and synchronous activities. Chart showing the total number of students who accessed and completed synchronous and asynchronous activities on the Moodle learning management system. The pretest and posttest are synchronous while the assignment and course evaluation are asynchronous

Nine hundred thirty-three students (49% female and 51% male) and 22 lecturers took part in the study and were enrolled in the research course. Our records show that 65% (*n* = 613) of students attempted the pretest, but only 53% (323) answered all questions. Also, we provided a posttest at the end of the module, but only 62% (579/933) of students accessed it. Of these students, only 45% (266/579) completed the posttest exam. We also tracked the completion of two asynchronous activities: the submission of an assignment and the completion of an evaluation of the course. 60% (563/933) of the students submitted the assignment and 54% (510/933) completed the course evaluation (Fig. 1). Students who were not complete the synchronous and asynchronous activities through the LMS were required to submit them to their course coordinators via email.

### Value and quality of the course

**Fig. 2:**
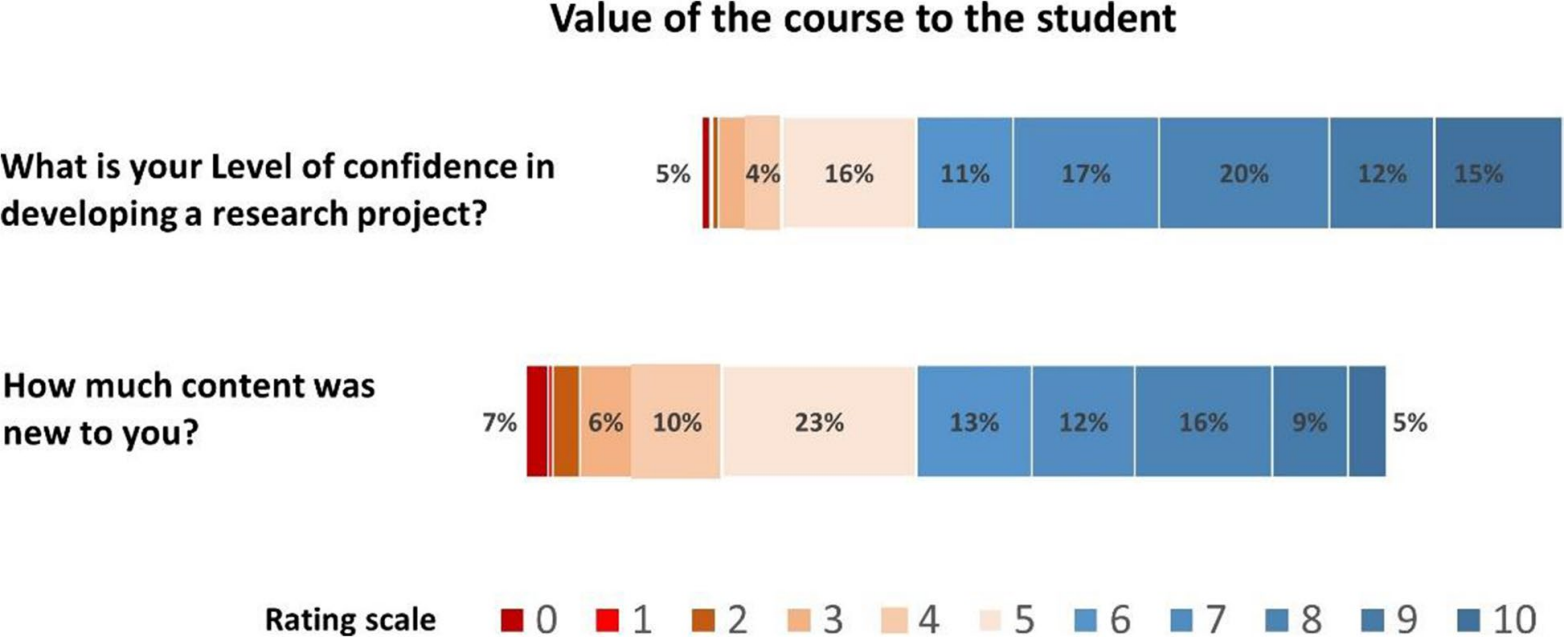
Value of the course to the student. Chart showing students ratings for 2 questions to the overall value of the course. Students were required to rate each question on a scale of 0-10. Ratings that were higher or equal to 6/10 are coloured in blue, indicating a positive response to the questions

**Fig. 3:**
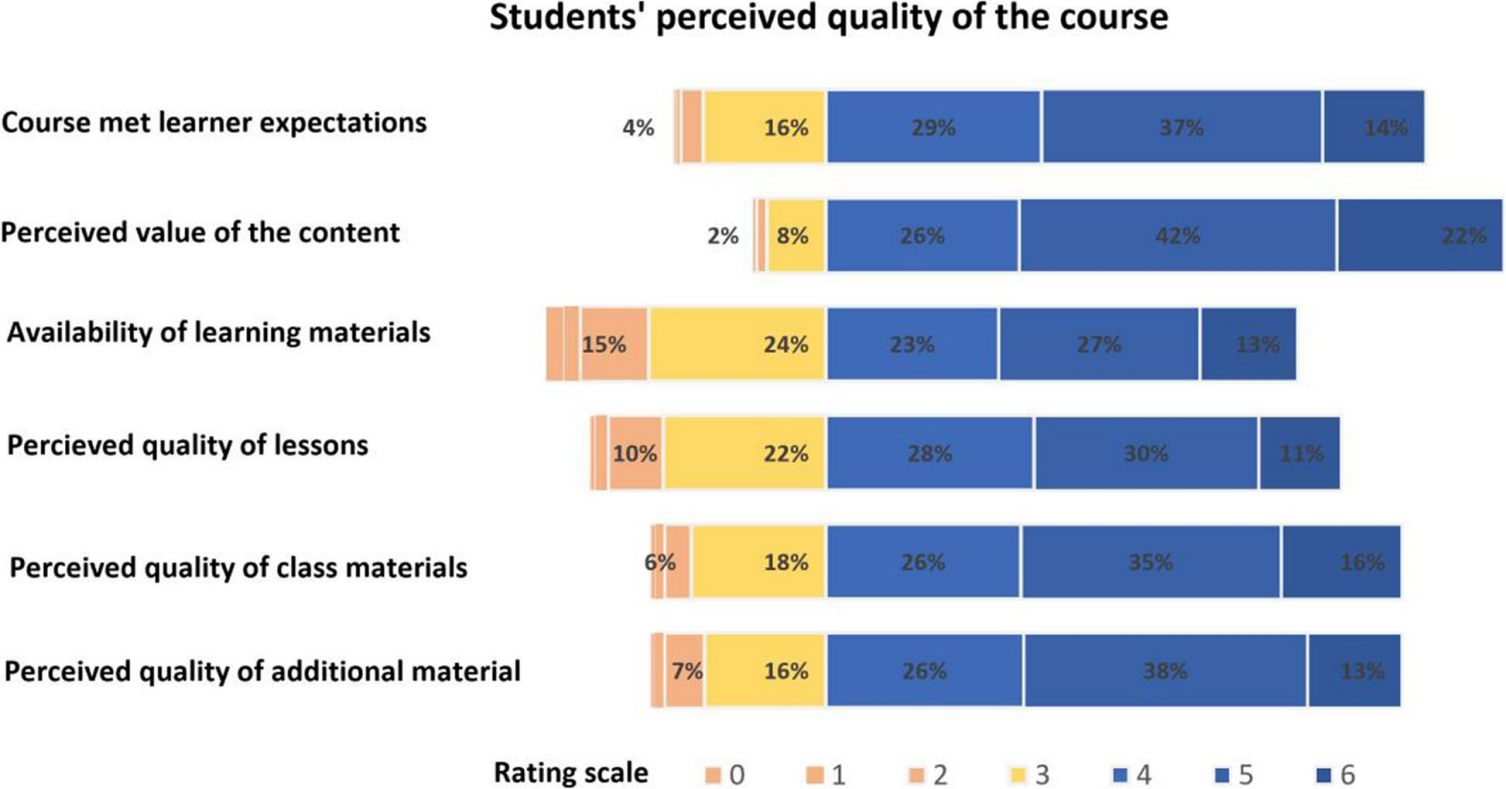
Student’s perception on the quality of the research course. Chart showing students’ ratings for 6 questions relating to the overall quality of the course. Students were required to rate each item on a scale of 0-6. Ratings that are higher or equal to 4/6 are coloured in blue, indicating a positive response to the questions

We asked students to rate the value of the course using a numeric-rating scale of zero to ten, with zero indicating an extremely low rating and ten indicating an extremely high rating. We also asked students to rate the quality of the course on an alpha-numeric scale of zero to six, with zero indicating an extremely low rating and six indicating an extremely high rating. A vast majority of students found the course to be valuable. Also, most students found the course to be of acceptable quality (Figs. 2 and 3).

### Themes reflecting students’ experiences on e-learning

We identified four themes related to students’ experiences of teaching and learning on the online research course. Each of the themes is discussed below.

### Understanding of the course

This theme encapsulates the student’s understanding of the course. Most of the students reported that the course had helped them develop competency in research.

*“It was a great learning experience from the first to the last session….and all sessions were equally useful I guess it’s because I had very little knowledge on research”* [Student on the course]

However, some students felt that the online mode of teaching was not appropriate for the delivery of some aspects of the course.

*“I did not understand the data management session very well. Will there be a recap when we resume physical learning?”*

Still, most students felt that the use of diverse technologies for teaching improved their understanding of the course.

“*I found the recorded sessions to be useful for revision and future reference. The slides and reference materials were also useful. I also enjoyed the virtual classes which allowed interaction between the teachers and the students*”

### Need for orientation to the e-learning platform

This theme encompasses the need to train students on the use of the e-learning platform. The students reported several personal factors that made it difficult to access the Moodle LMS. Indeed, some students suggested that faculty should train students on how to use the Moodle platform before teaching. Other students pointed out that they spent the first week of the course learning how to navigate the e-learning platform.

### The feeling of being overwhelmed

This theme covers students’ feelings of being overwhelmed because of having a lot of learning content to cover and online classes to attend. Some students reported they were still working and did not have time to attend all classes.

“*General presentation of the course was good. However, I am a distance learning student and I had difficulty attending all the lessons because I had responsibilities at my workplace.”*

The strongest expression of this feeling was in relation to the duration of the course. Most students felt that the contact time with lecturers was inadequate. Some students suggested the college should extend the course by 1 week.

*“Research sessions were good and well presented. l do appreciate your effort. Well, done. But l have a few issues to raise, one the workload was too much and the time allocated was minimal, l think given three weeks could be much better for us to have enough time to tackle the review questions and activities.”*

### Technological challenges

The theme encapsulates technological challenges faced by students during the online course. Most students reported they did not have access to the e-learning platform during synchronous activities.

*“I did not finish my posttest exam yesterday as the system had malfunctioned due to technical issues. I only attempted 20 questions before this problem arose.”* [Student on the course]

Many students reported they could not download learning resources that were in PDF or PPT format. Other students reported dead links to additional resources that were provided on the course. Students suggested lecturers should use different technologies to share learning resources because the Moodle platform was unreliable.

*“The provision of notes in PowerPoint form on the platform was a good idea. But the problem is they were not always available… I mean, today the notes are there, but the next day they are unavailable.”* [Student on the course]

Also, some students suggested lecturers should use different technologies to share learning resources because the Moodle platform was unreliable. The students involved in this course were grateful for the wealth of material and perspectives they acquired in this course.

*“On my side, every topic is useful to me because l will implement the knowledge l have acquired to write my research, which every step learnt is important.”* [Student on the course]

*“It was a great learning experience from the first to the last session….and all sessions were equally useful I guess it’s because I had very little knowledge on research”* [Student on the course]

### Themes reflecting faculty experiences on e-learning

Faculty experienced constraints such as insufficient technical skills, time constraints on already overloaded staff, the need for incentives and discomfort with the sudden requirement to share content online, including privacy concerns. These were overcome through a team approach and faculty feedback was generally positive:

‘*It has been a great learning experience…let’s all celebrate this milestone in the history of KMTC…we have contributed to the success (of the research course)…thanks to the team leadership*’ [Lecturer on the course]

‘*That was a great team, and I am glad to be part of it*’ [Lecturer on the course]

*‘The organization and coordination skills were admirable*…’ [Lecturer on the course]

## Discussion

The effectiveness of the e-learning approach varies from context to context. It often requires sustained learner motivation, digital literacy and significant investment by the host institution [16]. In this course, we provided a mix of synchronous and asynchronous learning opportunities to 933 students. Students enrolled in the course felt that this approach enhanced their understanding of the course. Indeed, Ruggeri et al. [16] considers this approach as an essential factor for the success of e-learning. After completing the e-learning course on various aspects of research, students’ knowledge, readiness and confidence to conduct a research project increased. Other studies have given comparable results, which show that the use of e-learning in medical education enhances knowledge acquisition, especially for topics that are complex [10, 17].

The e-learning approach allows institutions to maximise the use of resources [4]. In this study, our institution quickly assembled a team of lecturers from different specialties, campuses, and regions to teach the online research course. This approach allowed a small group of lecturers (22) to teach a large group of students (933). In contrast, a face-to-face approach would have required twice the number of lecturers (44) to teach the same group. Also, our study findings are consistent with other studies that reported a reduction in workload for teachers who use the e-learning approach [6, 11]. Some studies have reported an improvement in technological skills for lecturers who take part in e-learning [18]. In our study, lecturers reported positive experiences, such as increased efficiency, effectiveness and interdisciplinary collaboration.

Previous studies have identified technological challenges as a major barrier to e-learning [11–14]. These challenges are more pronounced in LMICs as compared to upper and middle-income countries [7]. Educational institutions that transitioned from face-face teaching to eLearning during the COVID-19 pandemic in LMICs appear to have faced technological barriers [11, 12, 18]. In contrast, studies by Reid et al. [19] (United Kingdom) and Song et al. [20] (China) reported an easier transition to e-learning during this period. Specifically, Song et al. [20] reported that students and teachers were satisfied with the implementation of e-learning during the COVID-19 pandemic. Our study findings show that lecturers and students faced technological challenges when accessing the e-learning platform. Also, some students pointed out that they could not navigate the platform because they lacked the requisite IT skills. Therefore, there is need to provide such training to enhance student learning experience. The technical challenges experienced in this online course underscore the need for strong institutional support, as highlighted in other literature [1]. To promote e-learning, there is a need to improve institutional readiness and support (e.g., provide ICT support, developing skills of lecturers, provide access to computers, increase bandwidth and procure a videoconferencing solution).

The study has several strengths. We used routine educational data, collected during the course, which reduced the risk of recall bias. We also included data from all participants in the course, as enrolled on the e-learning platform, which reduced the risk of selection bias. There is a risk of social desirability bias, where participants may give only positive feedback on their experiences. However, we used triangulation of data sources and tools to mitigate.

## Limitations

The generalizability of the results is limited because we only included students from Kenya Medical Training College. However, we minimised the potential for selection bias by including all higher diploma courses on all campuses. Also, student participation in the research course was mandatory. We did not assess the impact on students with disabilities, and we did not collect data on the costs of training - this evidence would be helpful to develop a robust understanding of the potential gains of e-learning.

To overcome weaknesses of this study, we are currently designing a randomized controlled trial in which we will investigate the effectiveness of an e-learning research course in an intervention group, compared to a control group that uses the traditional face-to-face mode of teaching and learning.

## Conclusion

In our experience, the use of e-learning for teaching research has been feasible and successful. Critical thinking, creativity, collaboration, communication, technological investment and institutional readiness are required in the successful adoption of e-learning for such large classes. There is a need to ensure that its integration takes into consideration the local context and accounts for the specific needs of learners and faculty [21]. Other educators and researchers might find this experience useful. Future studies could investigate the long-term effects of the course on the research practices of the students.

## Data Availability

The dataset is available from the corresponding author on reasonable request to the head of institution.

## Abbreviations

ICT: Information, communication and technology
KMTC: Kenya Medical Training College
WHO: World Health Organization
LMS: Learning management systems
LMICs: Low- and middle-income countries
